# Comment on “Elevation of transaminases after MMP® session with methotrexate for alopecia areata treatment - how much do we know about the risks of systemic absorption of the technique?”^☆^

**DOI:** 10.1016/j.abd.2023.05.002

**Published:** 2023-10-05

**Authors:** Samir Arbache, Sergio Henrique Hirata

**Affiliations:** a DermoCentro Clinic, São José dos Campos, SP, Brazil; b Department of Dermatology, Universidade Federal de São Paulo, São Paulo, SP, Brazil

Dear Editor,

We read with interest Nogueira’s et al. recent case report.^1^ This is an important contribution to the limited evidence about the safety of drug delivery techniques.

As an experienced and enthusiastic user of the MMP® technique, I have a few considerations:

1) Although evidence derived from case reports is considered low, nevertheless it is important to raise questions in different scenarios.
2) Changes in liver enzyme levels are common in clinical practice and can be caused by many factors including physical activity, use of drugs (e.g., acetaminophen) or alcohol, and viral infections.^2^
3) MMP® is a drug delivery technique that uses microneedles and dermo pigmentation equipment. Tattoos are unequivocal evidence of injection and absorption. The main advantages of this technique are the uniform drug distribution in the dermis, without bolus formation (Fig. 1), and increased substance dispersion due to shear stress and turbulent whirling^3^ caused by Newton’s law of attrition.

**Figure 1:**
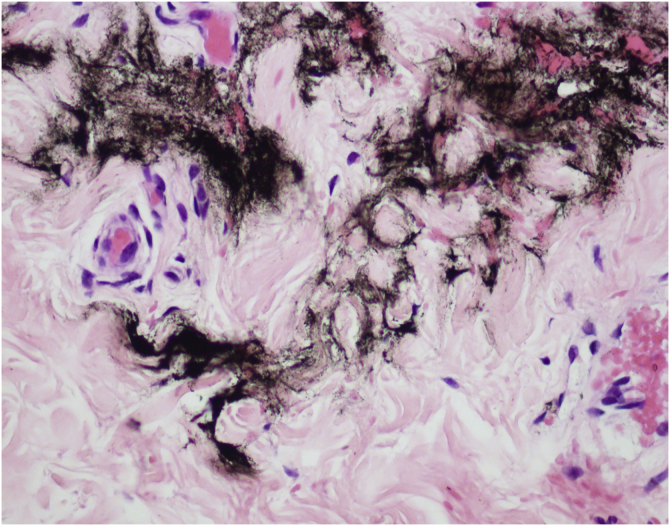
Drug delivery with MMP® technique. This is a cross section of the reticular dermis demonstrating ink between the collagen fibers, without bolus formation
4) Drug absorption delivered through MMP® occurs predominantly through the lymphatic channels of the skin^4^ and not the subjacent blood vessels.
5) MMP® is the only drug delivery technique with published protocols that allow the quantification of the drug density injected in the dermis,^5^ offering safety parameters to the dermatologist.
6) Since the procedure was done in an ophiasis region (423 cm^2^), using a saline solution (estimated density 1.000.000 µg/mL) and the medication contained 25 mg/mL of the active ingredient, based on published protocols we calculate that 390.907 µg (0.390907 mg) of the methotrexate solution was injected in the dermis^5^ (Video 1), which corresponds to 9.8 mg of methotrexate.

Considering the aforementioned facts, and the lack of other reports of possible drug toxicity related to methotrexate MMP® drug delivery, we must consider an alternative hypothesis for the findings described by Nogueira et al.^1^ It is possible that this patient’s mild transient increase in transaminases may have occurred by chance or have been caused by other factors unrelated to methotrexate MMP® drug delivery?

## Financial support

TRADERM, a company that commercializes tattoo supplies.

## Authors' contributions

Samir Arbache: Conceptualization; data curation; writing-original draft; and writing-review and editing.

Sergio Henrique Hirata: Conceptualization; data curation; writing-original draft; and writing-review and editing.

## Conflicts of interest

MMP®, a registered trademark in Brazil, the United States, and Europe, grants free use exclusively to dermatologists who are members of the Brazilian Society of Dermatology and equivalent associations in the world. Dr. Arbache owns the company that commercializes the supplies used for MMP and is part of a team of professionals who train Brazilian dermatologists in to use of this technique.
